# Distinct Roles of Broadly Expressed Repressors Support Dynamic Enhancer Action and Change in Time

**DOI:** 10.1016/j.celrep.2019.06.063

**Published:** 2019-07-23

**Authors:** Theodora Koromila, Angelike Stathopoulos

**Affiliations:** 1 Division of Biology and Biological Engineering, California Institute of Technology, 1200 East California Blvd., Pasadena, CA 91125, USA; 2 Lead Contact

## Abstract

How broadly expressed repressors regulate gene expression is incompletely understood. To gain insight, we investigated how Suppressor of Hairless—Su(H)—and Runt regulate expression of bone morphogenetic protein (BMP) antagonist *short-gastrulation* via the *sog_Distal* enhancer. A live imaging protocol was optimized to capture this enhancer’s spatiotemporal output throughout the early *Drosophila* embryo, finding in this context that Runt regulates transcription initiation, Su(H) regulates transcription rate, and both factors control spatial expression. Furthermore, whereas Su(H) functions as a dedicated repressor, Runt temporally switches from repressor to activator. Our results demonstrate that broad repressors play temporally distinct roles and contribute to dynamic gene expression. Both Run and Su(H)’s ability to influence the spatiotemporal domains of gene expression may serve to counterbalance activators and function in this manner as important regulators of the maternal-to-zygotic transition in early embryos.

## In Brief

The study by Koromila and Stathopoulos defines roles for broadly expressed repressors Suppressor of Hairless and Runt in supporting dynamic gene expression in early *Drosophila* embryos. Using live *in vivo* imaging and quantitative analysis of reporter outputs, they demonstrate that these factors support different functions, affecting transcription initiation versus levels.

## Graphical Abstract

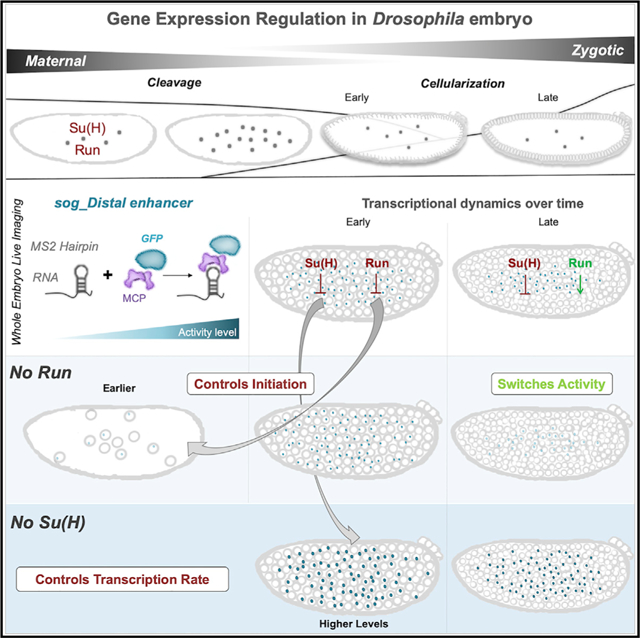

## INTRODUCTION

One of the major challenges of the modern genomics era is to better understand how gene expression is regulated to support spatiotemporal outputs that change over the course of development. The early *Drosophila* embryo has served as a paradigm for how enhancers control patterning and has also demonstrated that the patterning process is dynamic. It is known that multiple, transiently acting enhancers act sequentially to support changing outputs of expression for some genes (e.g., Dunipace et al., 2013; Long et al., 2016; Perry et al., 2012), whereas other genes are controlled by enhancers that act over a longer period and support changing spatial outputs over time. For example, expression of the gene *short gastrulation* (*sog*) is driven by at least two co-acting enhancers that support temporally dynamic, overlapping outputs in the early blastoderm embryo for a prolonged period of time through gastrulation (Hong et al., 2008). Similar cis-regulatory systems that regulate spatiotemporal expression over the course of development are found in other organisms as well (Yuh and Davidson, 1996).

Dynamics associated with individual enhancers could relate to a changing landscape of transcription factors (TFs) binding to these sequences over time, for example, gain of new activator inputs, loss of repression, or both. For instance, our recent work showed that broadly expressed TFs, such as Suppressor of Hairless [Su(H)] and Runt (Run), act as repressors to limit both the spatial and temporal expression of enhancers, including those driving *sog* (Koromila and Stathopoulos, 2017). Our data supported the view that repressors either regulate the timing of action for different enhancers acting in series or, alternatively, influence the length of time a single enhancer is active to impact spatiotemporal outputs. However, the mechanism by which broadly expressed repressors act likely differs from that of well-characterized spatially localized repressors (reviewed in Ip and Hemavathy, 1997; Stathopoulos and Levine, 2005). However, Su(H) and Run’s mechanisms of action as broadly expressed repressors, including whether they act independently or coordinately, have not been determined.

Furthermore, input by broadly acting factors like Zelda (Zld) or Su(H)/Run may ensure the temporal regulation of the global maternal-to-zygotic transition (MZT) (Laver et al., 2015), which occurs during early embryonic development starting soon after fertilization, and these factors likely act earlier and distinct from deployment of spatially localized TFs. However, we hypothesized that Su(H) and Run may also allow an individual enhancer to change its spatial output over a prolonged period of time as well as to prevent ectopic expression outside of the proper context. Therefore, in this study, we set out to determine how broadly expressed TFs, Run and ubiquitously expressed Su(H), impact enhancer dynamics and whether these factors have equivalent functions.

## RESULTS AND DISCUSSION

### Su(H) and Run TFs Occupy *sog_Distal* Enhancer Sequence *In Vivo* and Modulate Gene Expression Output

Previous studies have shown that binding sites for Zld, Dorsal (DL), and Su(H) (Figure S1A) present within enhancers, *sog_Distal* or *sog_Intronic*, support *sog* expression (Foo et al., 2014; Liberman and Stathopoulos, 2009; Ozdemir et al., 2014). Zld and DL function as activators to support spatial expression such that in their absence the pattern either collapses to a thin ventrolateral stripe or is absent, respectively (Foo et al., 2014; Liberman and Stathopoulos, 2009). On the other hand, Su(H) was shown to act as a repressor that influences spatial outputs by serving as a counterbalance to DL- and Zld-mediated activation (Ozdemir et al., 2014). *sog_Distal* enhancer-driven *LacZ* reporter outputs were expanded, specifying a broader stripe in *Su(H)* mutants or when Su(H) sites were mutated within the enhancer sequence. Most recently, additional roles for Su(H) as well as for another broadly expressed repressor, Run, in supporting enhancer action were identified through analysis of a time series of carefully staged, fixed embryos (Koromila and Stathopoulos, 2017). To provide further insight into the mechanisms used by these repressors to support dynamic gene expression, we carefully analyzed how Su(H) and Run mutations affect temporal gene expression.

**Figure 1.**
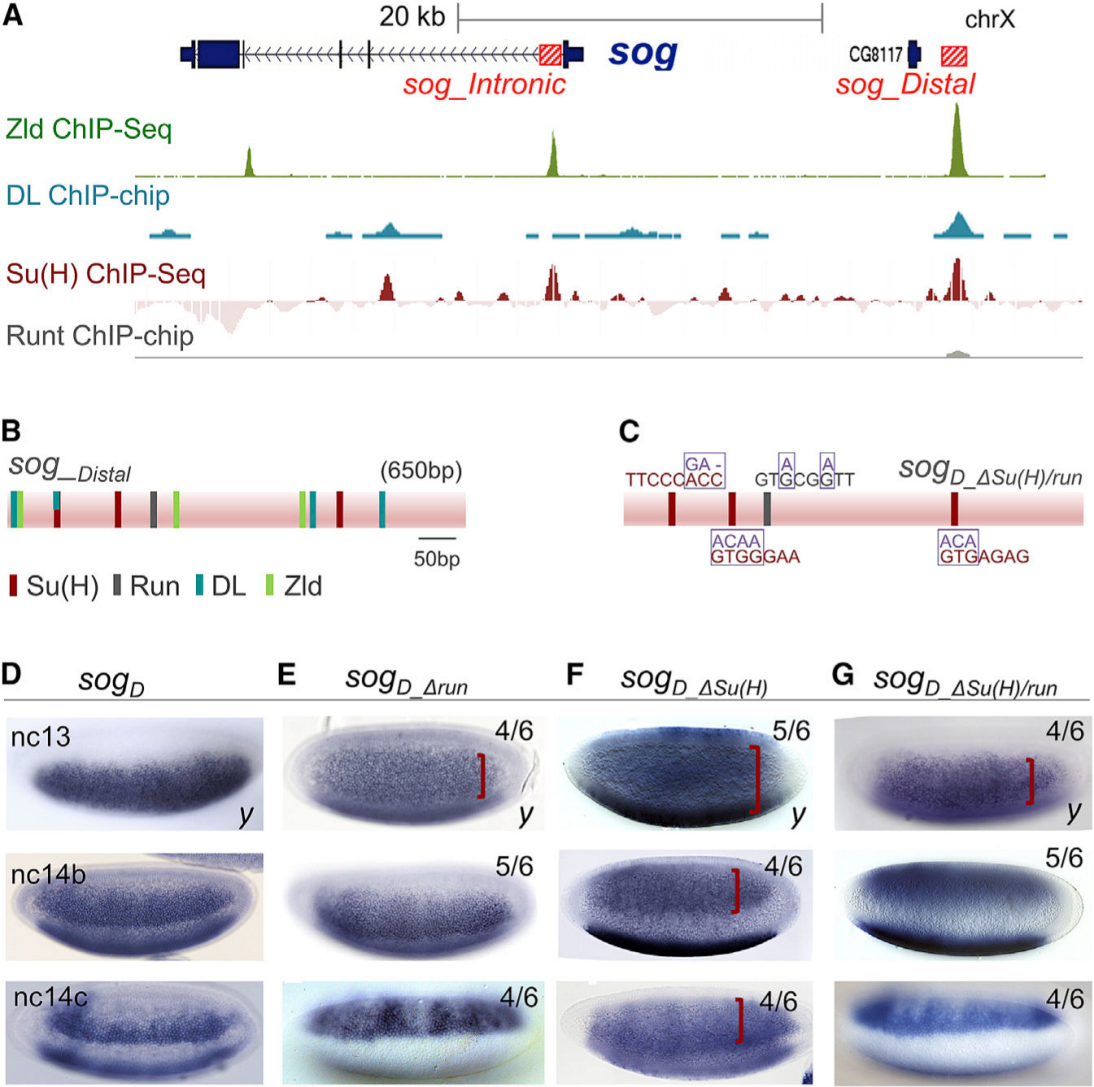
ChIP Detects the Occupancy of Su(H) and Runat *sog_Distal* Enhancer *In Vivo*, whereas Mutation of Predicted Binding Sites in Reporter Constructs Affects Gene Expression Outputs in Fixed Embryos (A) ChIP-defined occupancy of Zelda (Zld), Su(H), and Runt transcription factors at the *sog* locus as detected previously (Harrison et al., 2011; MacArthur et al., 2009; Ozdemir et al., 2014) showing binding of all three factors to *sog_Distal* enhancer located ~12 kb upstream of the promoter, but binding of only Zld and Su(H) to the *sog_Intronic* enhancer located just downstream of the promoter in the first intron. (B) Schematic of *sog_Distal* enhancer sequence of ~650 bp in length showing location and number of matches to Su(H), Run, DL, and Zld consensus binding motifs. (C) Sequence information relating to the one Run (gray) and three Su(H) (red) binding sites found by matching to consensus sequences and the changes introduced upon mutagenesis (purple). (D–G) Embryos at stage nc13, nc14b, and nc14c stained by *in situ* hybridization using intronic *yellow* riboprobe to assay reporter expression supported by constructs *sog_Distal* (D), *sog*_*D*_*_*Δrun (E), *sog*_*D*_*_ΔSu(H)* (F), and *sog*_*D*_*_ΔSu(H)/run* (G). Red brackets show the expanded *sog*_*Distal* expression pattern associated with mutant constructs. In this and subsequent panels, lateral or ventrolateral views of embryos are shown with anterior to the left and dorsal side up, unless otherwise noted. See also Figure S1.

To start, we used an ectopic expression assay finding that Su(H) represses activity of both enhancers, while Run represses *sog_Distal* but has no effect on *sog_Intronic* (Figures S1B and S1D). These results confirm evidence provided by chromatin immunoprecipitation (ChIP) data that both factors influence *sog_Distal* expression, whereas only Su(H) impacts *sog_Intronic* (Figure 1A). In support of these findings, matches to the Su(H) consensus binding sequence (RTGRGAR) were identified within both enhancers (Ozdemir et al., 2014), whereas only a single match to the Run consensus binding motif (ACCGCA) was identified in *sog_Distal* enhancer and no match was found within the *sog_Intronic* enhancer (Figures 1B and S1E). Previous studies have identified the sequence ACCRCA as a consensus Runx (mammalian ortholog of Run) binding motif (Melnikova et al., 1993). The six amino acids that are the primary determinants of Runx1 DNA binding specificity are all identical in the *Drosophila* Run amino acid sequence, strongly suggesting that binding sites recognized by Run will conform to the Runx consensus (Lewis et al., 1999).

To provide additional insight into Su(H) and Run’s mechanism of action, we focused on assessing their roles in supporting dynamic *sog_Distal* gene expression output. A *sog*_*Distal* reporter was constructed in which the enhancer sequence was placed upstream of a heterologous promoter from the *even-skipped* gene (*eve.p*) driving expression of a bipartite reporter, containing *yellow* gene and MS2 RNA stem-loop encoding sequences, to permit analysis of outputs in fixed as well as live embryos, respectively (see STAR Methods). Through mutagenesis experiments, the influence of predicted binding sites for Su(H) and Run on *sog_Distal* enhancer-driven gene expression was assayed, paying close attention to phenotypes displaying a changed spatial pattern or timing through a comparison of reporter gene outputs.

The *Drosophila* embryo develops quickly, as a syncytium, for the first 14 nuclear divisions before cellularization occurs. Nuclear cycle (nc) 14 is longer than the preceding cycles and lasts ~45 min. Therefore, we assayed gene expression progression within fixed embryos during nc13 and nc14, using three ~10-min intervals to assay the latter cycle: nc14a, nc14b, and nc14c. *In situ* hybridizations using a riboprobe to the *yellow* gene, which serves as reporter in fixed samples, provided insight into the dynamic expression supported by the enhancer both spatially and temporally when multiple embryos representing a time series were compared. For example, *sog_Distal* reporter expression changes over time, with the pattern encompassing broad expression during nc13 and nc14a and decreasing to a more narrow ventrolateral stripe at later stages, nc14b and nc14c (Figure 1D).

Matches to consensus binding sites for Su(H) or Run were identified within the *sog_Distal* enhancer sequence (Figure 1B) and mutated (Figure 1C), taking care not to destroy overlapping binding sites (Figure S1A), when identifiable, or to introduce known binding sites for other relevant factors. A previous study examined reporter expression in fixed embryos containing mutated enhancers to determine the effect of mutation of three Su(H) binding sites within the *sog_Distal* enhancer [*sog*_*D*_*_*Δ*Su(H)*], finding that this leads to an expansion of the pattern at nc14 (Figure 1F; Ozdemir et al., 2014). We noticed that the pattern is expanded not only in nc14b,c but also during nc13 (Figure 1F, top). Similarly, mutagenesis of the single Run site present in this enhancer (*sog*_*D*_*_*Δ*run*), a perfect match to the consensus and therefore likely to be of high affinity, also leads to expansion of the pattern during nc13 (Figure 1E, top). In contrast, in nc14, the pattern of *sogD_*Δ*run* appears relatively normal compared to the wild-type (Figure 1E, bottom; Koromila and Stathopoulos, 2017). These fixed embryo results support the view that during nc13 both these TFs function as repressors in the context of *sog_Distal*, as when their inputs are abolished, the reporter outputs expand spatially. At later stage nc14, the roles of these factors likely diverge. At nc14, the expression domain increases upon loss of Su(H) input, suggesting that Su(H) continues to support repression, whereas no change is observed upon loss of Run, suggesting this factor no longer supports repression at this stage. In the double mutant [*sog*_*D*_*_*Δ*Su(H)/run*], the pattern was expanded at nc13, as for either Su(H) or Run single mutants; however, no significant increase was observed at nc14b or nc14c, a trend similar to the Run single mutant (Figure 1G). Temporally changing roles for factors can be inferred by studying a time series of fixed embryos, but to provide more definitive evidence we turned to live imaging.

### The MS2-MCP Imaging System Can Be Used to Monitor *sog_Distal* Gene Expression Dynamics

Live imaging experiments offer the capacity to analyze gene expression dynamics with increased temporal resolution and linear quantification. To assess these mutant enhancer phenotypes systematically, we developed a quantitative approach to measure the spatiotemporal outputs of enhancer-driven MS2-yellow reporter constructs as captured by *in vivo* imaging to provide information about the timing, levels, and spatial domains of expression.

**Figure 2.**
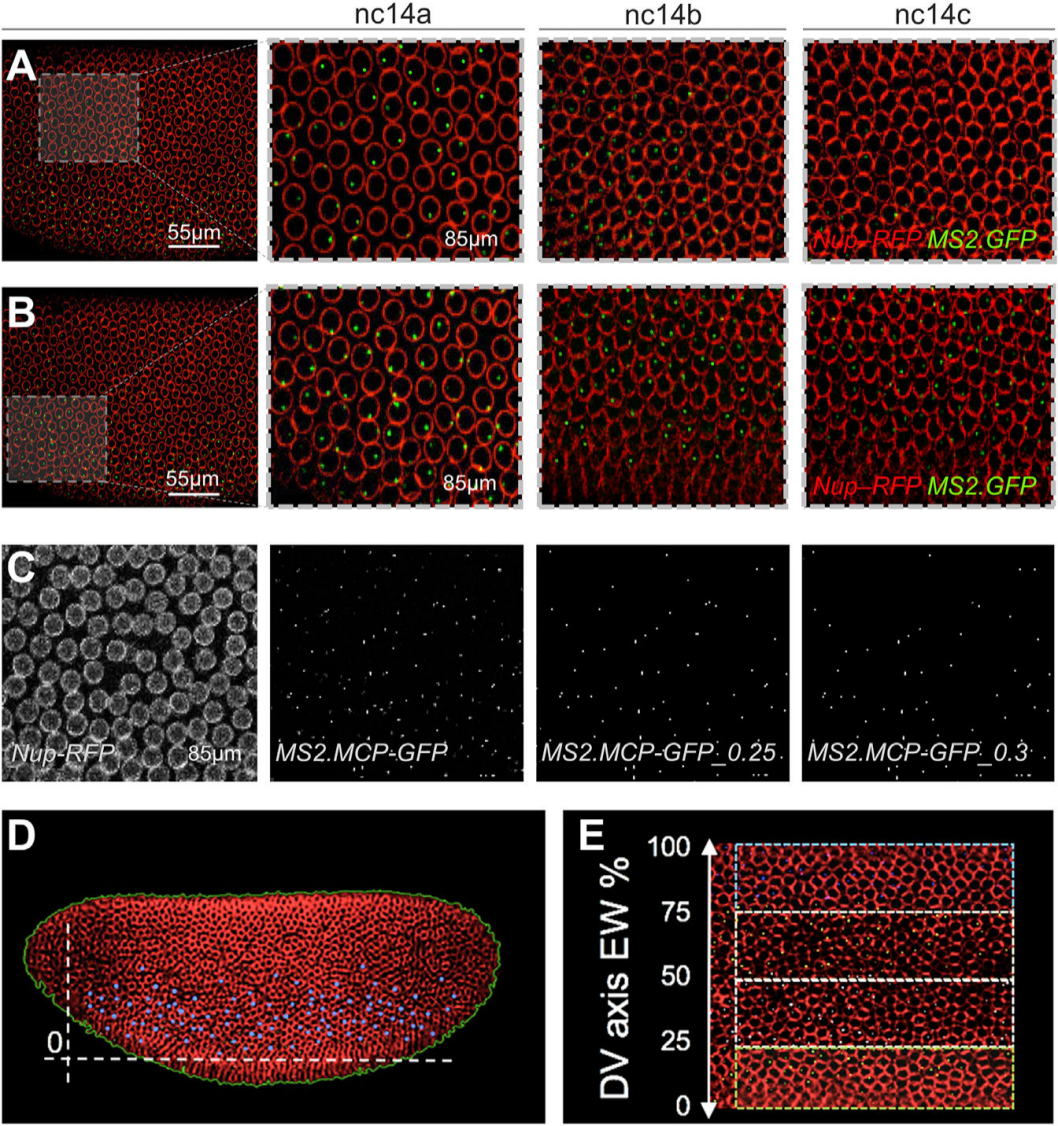
Live Imaging of *sog_Distal* Reporter Gene Expression Is Supported by the MS2-MCP System, and Quantitative Analysis Can Provide Information Regarding the Spatiotemporal Dynamics of Reporter Expression across the Full Embryo (A and B) Snapshots from a single imaging session of *sog_*Distal *MS2-yellow* transgene highlighting either lateral (A) or ventral (B) views in which nascent transcripts were visualized in the nucleus by GFP fluorescence associated with detection of dots, representing MS2-MCP identified nascent transcripts (green), and RFP-fluorescence associated with nuclear Nup-RFP (red). Dashed boxes within panels to the far left represent different vantage point positions within imaged embryos, with magnified view shown to the right, over the course of nc14. (C) Background thresholding of the raw *sog* Distal MS2.MCP expression (MS2.MCP-GFP) localized within Nup-RFP marked nuclear membranes (left) using two different baseline thresholds (BTH = 0.25 and 0.3) allows identification of nascent sites of transcription and eliminates background noise. (D) After estimation of embryo boundaries (green perimeter line) and identification of nascent transcription dots (blue), the ventral boundary of expression was also defined based on where the signal decreases to a level that passes below the baseline threshold (BTH; see also Figure S2D). (E) To support quantitative analysis of data along the DV axis, relative positions from 0 to 100 along the embryo width (EW) were used in order to facilitate comparison of different embryos. See also Figure S2 and Video S2.

Using the same transgenic lines, we conducted live imaging of GFP-positive signal associated with the MS2 stem-loop reporter sequence just upstream of the *yellow* gene in the reporter construct (see Figure S2A; Bertrand et al., 1998). This MS2 cassette contains 24 repeats of a DNA sequence that produces an RNA stem loop when transcribed. The stem-loop structure can be specifically bound by the phage MS2 coat protein (MCP). MCP fused to GFP and bound to MS2-containing transcript (i.e., *sog_Distal.MS2*) produces a strong green signal within the nuclei of *Drosophila* embryos at the site of nascent transcript production. During active transcription, these fluorescent spots are composed of several fluorescently labeled mRNAs (e.g., Figures 2A and 2B) (Garcia et al., 2013; Lucas et al., 2013). The MCP-GFP protein also is maternally provided, allowing ample time for maturation of the fluorophore in early embryos, and nuclear localization sequences native to the GFP protein are mutated to decrease the background signal associated with accumulation of unbound fusion protein within the nucleus (Garcia et al., 2013). In this system, the nuclear GFP signal is only observed as a single dot for every nucleus corresponding to nascent transcription of the one copy of the MS2-containing reporter transgene site integrated into the genome (see STAR Methods). Furthermore, the nuclear periphery is marked by a fusion of RFP to nuclear lamin protein (Nup-RFP) (Lucas et al., 2013).

The imaging protocol was optimized to provide spatial information across the entire dorsal-ventral (DV) axis of embryos with the fastest temporal resolution that also retains embryo viability (see STAR Methods). In brief, embryos were imaged continuously over the course of 2 h at an interval of ~60 s per scan, capturing complete lateral views at a depth of 50 um. Importantly, this imaging protocol is not phototoxic to embryos, as the viability of the imaged embryos was confirmed by checking that they hatch ~24 h later. Several studies have used a similar imaging approach to study gene expression in *Drosophila* embryos (e.g., Garcia et al., 2013; Lucas et al., 2013); however, the majority has focused on analysis of transcriptional responses through the assay of small regions of expression within embryos. Imaging smaller domains allows for increased temporal resolution of ~20 s per time point (e.g., Ferraro et al., 2016), but also can limit the spatial information that can be gathered. The expression pattern supported by *sog_Distal* is so dynamic that focused imaging on one small area of the embryo is not sufficient to describe the full spatiotemporal response of the enhancer output. For example, if the focus is directed to one small region in the lateral embryo, expression is detectable at earlier time points (i.e., nc14a and nc14b) but not later ones (i.e., nc14c), because the expression boundary shifts ventrally out of the field of view (Figure 2A). On the other hand, with a ventrolateral window of focus of the same embryo, expression is detectable at all time points (Figure 2B) but does not capture the dynamic expression pattern in more dorsal regions (e.g., Figure 2A).

Because spatial outputs likely change in time across the embryo for many gene expression patterns, we developed an image-processing approach to collect detailed information in both time and space by capturing a full lateral view. Image processing was used to extract data from videos (see STAR Methods). In brief, segmentation scripts were used to identify GFP-positive dots within Nup-RFP-labeled nuclear domains using an empirically chosen background threshold (BTH) setpoint (Figure 2C) and data analyzed in time (in terms of nc stage) or space using relative units of embryo width (EW) corresponding, approximately, to the DV axis position (Figures 2D and 2E).

### Onset of *sog_Distal* Gene Expression Is Regulated by Both Run and Su(H), but Only Su(H) Influences the Levels of Expression Output

**Figure 3.**
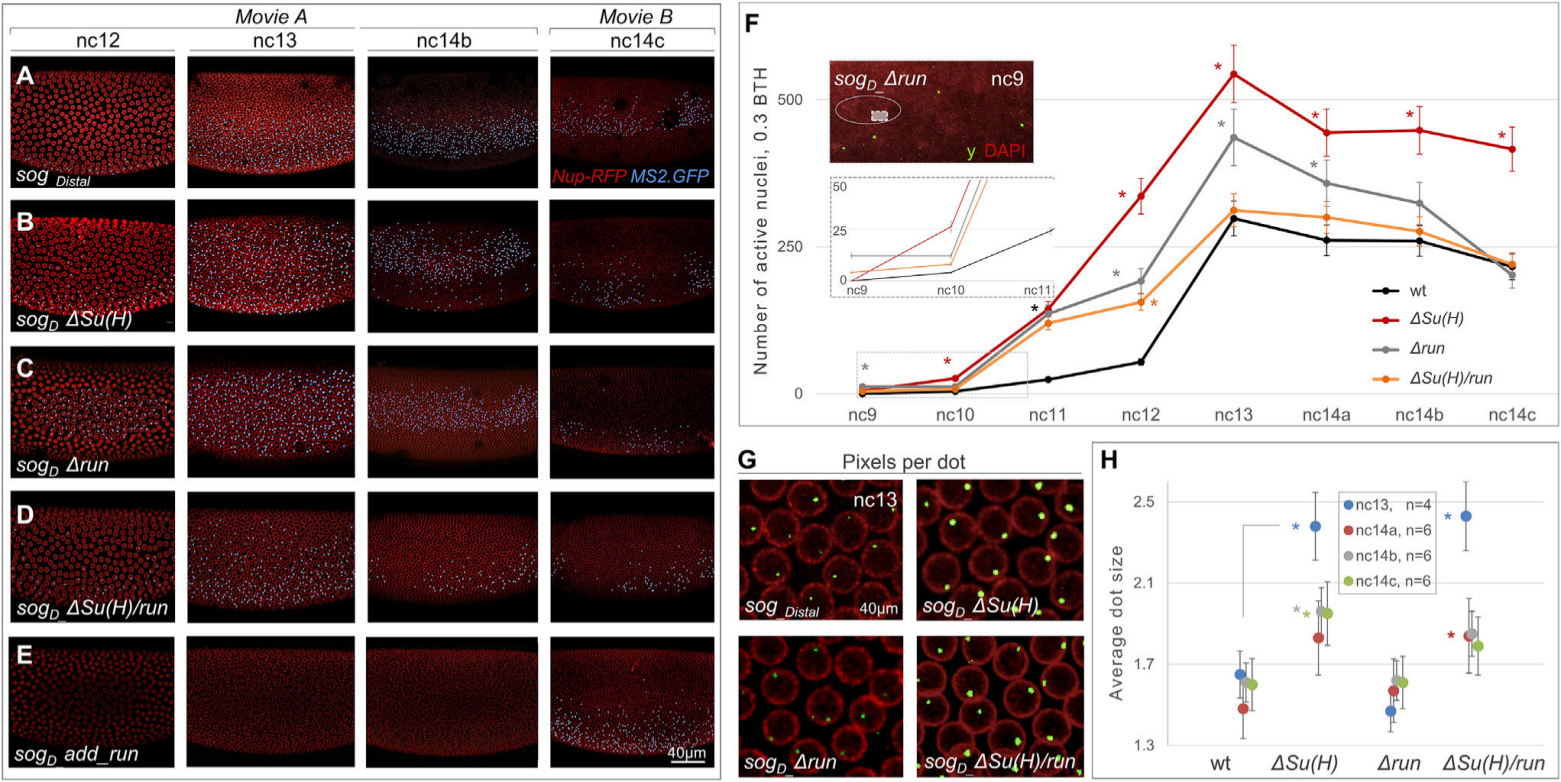
Quantitative Analysis of Imaging Data Provides Insight into the Initiation of Transcription as well as Levels of Transcription Associated with *sog_Distal* Enhancer Variants over Time (A–E) Stills from representative videos of the four indicated *sog_Distal MS2-yellow* reporter variants at four representative time points: nc12, nc13, and nc14b (stills from early blastula videos; Videos S1A, S2A, S3A, and S4A) and nc14c (stills from late blastula videos; Video S5B). To eliminate phototoxicity, embryos were imaged for a maximum of 2 h (nc9 to nc14b, Videos S1A, S2A, S3A, and S4A; nc14a-gastrulation, Video S5 B). Blue dots for each construct, wild type (A) or mutant (*sogD_ΔSu(H)* in B, *sogD_Δrun* in C, *sogD_ sogD_ΔSu(H)/run* in D, and *sogD add_run* in E), indicate GFP_+_ nascent transcripts labeled by the MS2-MCP system after thresholding was applied and remaining signals identified by the Imaris Bitplane software, for visualization purposes only. Scale bar represents 40 μm. (F) Total number of active nuclei (i.e., MS2-MCP signal after background thresholding; BTH = 0.3) per embryo averaged for all stills per time point spanning nc9 through nc14c. Moreover, such data were obtained and averaged for three (nc9 to nc12), four (nc13) and six (nc14a, nc14b, and nc14c) representative videos of wild-type or mutant variant *sog_Distal* MS2-yellow reporter constructs (see STAR Methods). The error bars represent SEM. Asterisks refer to comparison with control (i.e., WT) at the same time point. Insets: (Top) Magnified snapshots of embryo containing *sogD_Δrun* construct stained by fluorescent in situ hybridization (FISH) using *yellow* intronic riboprobe and co-stained with DAPI (pseudo-colored red) detecting reporter expression at nc9. (Bottom) Magnified view of active nuclei counts plot at nc9 to nc11 shows that in both *sogD_Δrun* and *sog*_*D*_*_ΔSu(H)/run* constructs active nuclei were detected in videos as early as nc9 (N*Δrun* = 15 ± 3, and N*ΔSu(H)/run* = 7 ± 2), but not in the other two constructs. (G) Magnified view of indicated reporter constructs (40 mm) showing differences in size of nascent GFP positive dots associated with MS2-MCP imaging within nuclei (Nup-RFP marked). (H) The plot shows the average dot size (in terms of number of four-adjacent pixel defined clusters per cluster per nucleus) after application of BTH = 0.3; indicative of the rate of transcription as more transcripts result in larger dots, calculated for the indicated reporter at nc13 (blue; n = 4 embryos), nc14a (red; n = 6 embryos), nc14b (gray; n = 6 embryos), and nc14c (green; n = 6 embryos). Student’s two-tailed t test was applied and significance is designated by p < 0.05. The error bars represent SEM. Data points are offset for ease of visualization. See also Figures S2 and S3 and Videos S1, S2, S3, S4, and S5.

Videos were obtained of *sog_Distal* reporter or mutant variants and processed to extract data regarding GFP fluorescence, representing nascent transcripts, upon mutation of Run and/or Su(H) binding sites within the *sog_Distal* enhancer sequence (Figures 3A–3D, S2B, and S2C; Videos S1, S2, S3, and S4). For each construct, videos were obtained and the number of GFP-positive dots was counted throughout the embryos for each nuclear cycle (nc9 to nc14c) to obtain a measure of the initiation of transcription as well as the dynamics of gene expression (Figures 3F, S3A, and S3C).

Using our *in vivo* imaging approach on embryos bearing wild-type *sog_Distal* driving the *MS2-yellow* reporter, we first examined reporter expression live by comparing results with two different BTH setpoints (BTH = 0.25 and BTH = 0.3; data available for comparison in Figures 3F, S3E, S3I, and S3J) through the analysis of projected scans associated with videos encompassing time points from nc10 to nc14c. Whereas for the analysis of nc9, specifically, the estimated number of active nuclei was calculated by counting the numbers of MS2 dots for each individual slice, because of challenges in visualization as the nuclei have not yet completed migration to the periphery of the embryo (e.g., see Figures S3G and S3H). Using BTH = 0.3, nascent transcripts are detected as early as nc10, but this is apparent only for a small number of nuclei (N = 4 ± 1) (Figure 3F, see inset). Less stringent threshold setpoints (i.e., BTH = 0.25 or lower) were excluded, since the MS2-GFP signal was detected at nc9, representing false positives because *sog_Distal* is known to initiate expression at nc10 (Figures S3E and S3F; Foo et al., 2014). Using BTH = 0.3, therefore, we quantified the number of active nuclei throughout the embryo supported by *sog_Distal* at all time points, finding the number of active nuclei increases from nc10 to nc13 and then decreases from nc14a through nc14c (Figures 3A and 3F).

Analysis of *sog_Distal* mutant reporter-driven outputs also provided insight into the difference in roles of the TFs Run and Su(H). When the single Run binding site was mutated in the *sog_Distal* enhancer (i.e., *Δrun*), active nuclei were detected as early as nc9 (N = 10 ± 2; Figure 3F, see inset) in contrast to initiation at nc10 in wild-type. This suggests that Run input normally acts to regulate the timing of transcription. Furthermore, while the number of active nuclei supported by the *Δrun* reporter continues to increase until nc13, the levels of expression, as well as the active nuclei number, decrease during nc14 (Figures 3F and 3H). Specifically, the number of active nuclei drops significantly during the transition from nc14b to nc14c in *Δrun*, whereas a smaller change is observed in wild-type or other mutant constructs (Figures S3J). This drop is even more apparent using a more stringent threshold (BTH = 0.35; Figure S3B and S3D). We hypothesized that this drop in expression for the construct may relate to a change in role for Run. To test this idea, an additional Run binding site, a perfect match to the DNA-binding consensus sequence with all core cytosines included (Lewis et al., 1999), was introduced into the context of this enhancer (*sog*_*D*_*_add*_*run)* (Figures S1C and S3I; Video S5). In the *add*_*run* reporter construct, (Figure 3E), the addition of a single Run site in the *sog*_*D*_*_add*_*run* keeps output to a minimum until nc14b (Video S5; Figures 3E and S3I), and suggests that Run switches roles to support activation at nc14. In contrast, when the three Su(H) binding sites were mutated in the *sog_Distal* enhancer [i.e., *ΔSu(H)*], there was no change in the onset of gene expression, which initiated at nc10 as for wild-type (Figure 3F, inset). However, there was a significant increase in the number of active nuclei supported by the *ΔSu(H)* construct relative to that supported by the wild-type reporter at every stage measured from nc10 to nc14c (Figures 3B and 3F). Collectively, these results support the view that Su(H) functions as a dedicated repressor, whereas Run also functions as a repressor but only prior to mid-nc14.

In addition, surprisingly, we found that the mutation of both Su(H) and Run binding sites simultaneously (Δ*Su(H)/run*) is more similar to the mutation of Run alone (*Δrun*) in terms of the number of active nuclei supported (Figure 3F; BTH = 0.3), and this trend is even more apparent when a more stringent threshold is used (Figure S2C; BTH = 0.35). Furthermore, in the double mutant, transcription initiation was also observed earlier, at nc9, similar to the Run single mutant (Figure 3F, see inset) but this was only apparent with the relaxed threshold of BTH = 0.3 (Figure 3F, see inset; compare with Figure S3F).

However, particular phenotypes exhibited by the double mutant were similar to *ΔSu(H)* single mutants including the size of GFP-positive dots, which varies with the number of MS2-containing nascent reporter transcripts in the nucleus (e.g., Figure 3G). Average dot size was calculated using the average number of four-adjacent pixels per cluster per nucleus (i.e., definition of a dot) as a measure (Figure 3H), calculated for each of the specified time periods associated with each of the four assayed constructs. The double and *ΔSu(H)* single mutants both exhibit higher signal intensity (i.e., average dot size is increased above 2; Figure 3H), presumably due to a higher rate of transcription. In addition, this phenotype was most apparent at nc13 with less of a difference observed in nc14. Alternatively, *Δrun* pixel-dot ratios were comparable to wild-type at all four stages examined. The *ΔSu(H)/run* double-mutant phenotype is thus more similar to that of *ΔSu(H)* (i.e., epistatic to *Su(H)* single mutant) in terms of transcription rate (Figures 3G, 3H, and S2E); whereas, the phenotype of the double mutant was better matched to that of *run* single mutants in terms of the number of active nuclei (Figure 3F and S3C). Collectively these results suggest that Run and Su(H) have distinct functions: Run acts to regulate the initiation of *sog_Distal* enhancer-mediated transcription, whereas Su(H) regulates the its levels of expression.

### Dynamic Changes to the Width of Reporter-Driven Expression Outputs Are Also Associated with the Mutation of Su(H) or Run Binding Sites within the *sog_Distal* Enhancer

In addition, we devised a quantitative approach to assay the widths of the expression domains supported by the reporter variants. EW positions are not reflections of absolute position along the DV axis but provide a metric of the size of the expression domains along the axis in relative units, to permit comparisons between embryos and different reporter constructs. Differences in the lateral rotation of individual embryos were corrected for by introducing a y-axis shift to the data; this allowed patterns obtained from video replicates to be overlaid and demonstrated that imaging results are consistent between embryos of a given genotype (Figure S2D). For width measurements of *sog_Distal* reporter expression, we applied a smoothing curve and a threshold cutoff at 30% of the curve’s maximum (TH = 0.3; see Figure S2D, vertical dashedlines, and VideosS1, S2, S3, and S4). Furthermore, the ventral position of the stripe was defined by the ventral-most position at which the signal decreases to a level that passes below the threshold (see Figure S2D, horizontal dashed line). This boundary corresponds to where the Snail (or other) repressor would normally act to downregulate *sog* expression.

**Figure 4.**
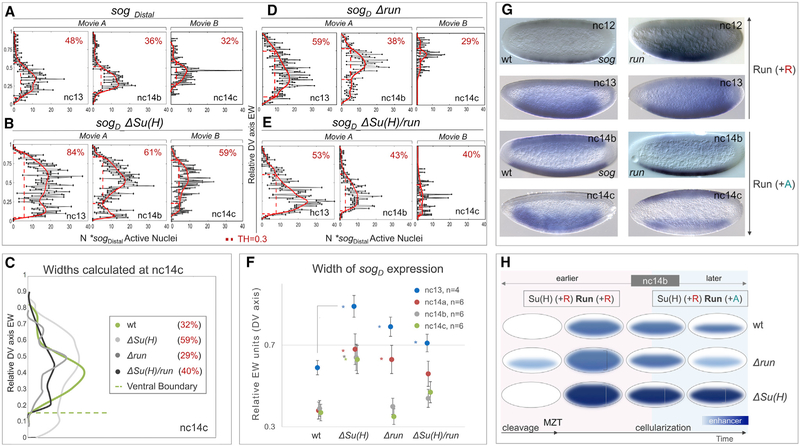
Quantitative Analysis of Imaging Data Also Provides Insight into the Width of Expression Domains Associated with *sog_Distal* Variants over Time (A, B, D, E) Plots of numbers of active nuclei, defined by counting dots (x axis), versus relative DV axis embryo-width (EW) position (y axis), as analyzed for representative stills from nc13, nc14b, and nc14c. To limit phototoxicity, embryos were imaged continuously for a maximum of 2 h. Therefore, to encompass the broad time course of nc9 to gastrulation, data from multiple embryos were obtained (nc9 to nc14b: Videos S1A, S2A, S3A, and S4A, early blastula embryo [embryo 1]; nc14a-gastrulation: Video S5B, late blastula embryo [embryo 2]). Black traces represent raw counts of active nuclei-dots of MS2-MCP signal (bins represent minimum of four dots) detected throughout embryos containing indicated constructs after projection of scans of particular time points were collapsed along the anterior-posterior (AP) axis and dots counted and binned across the DV axis (EW). The red trace for either wild-type (A) or mutant constructs (*sogD_ΔSu(H)* in B, *sogD_Δrun* in D, and *sogD_ sogD_ΔSu(H)/run* in E) represents normalization after application of a smoothing curve. Widths of reporter expression were defined as EW distance at 30% (TH = 0.3) of the signal (red vertical dashed line; values displayed in the top-right corner of each graph) (see STAR Methods). (C) Representative overlaid quantification curves of embryos for four constructs at nc14c (see A, B, D, and E above). Similarly, the x axis corresponds to the number of active nuclei, while the y axis indicates position along DV axis in relative EW units. Ventral boundary (gray dashed line) position of each construct is defined as the most ventral position that intersects with background threshold at nc13. (F) Small-scale statistical analysis of widths calculated at four comparable time points (nc13, nc14a, nc14b, and nc14c) for the indicated given number of embryos (n; see key) for each of the four constructs. Student’s two-tailed t test was applied and significance is designated by p value of < 0.05. Error bars represent SEM. Data points are offset for ease of visualization. (G) Loss-of-function *run* mutant embryos at nc12, nc13, nc14b, and nc14c stained by *in situ* hybridization using an intronic *sog* riboprobe to examine the effects on endogenous *sog* expression, demonstrating similar relation of Run to endogenous *sog* expression. (H) Summary schematic showing that Su(H) functions as a dedicated repressor, whereas Runt temporally switches from repressor to activator in time. Changes in domain of expression or levels of expression supported by results are indicated. In particular, *Δrun* mutants initiate transcription earlier at nc9, whereas *ΔSu(H)* mutants lead to an increase in levels of transcription and expansion of the spatial domain of expression. Furthermore, in the context of the *sog*_*Distal* enhancer, we propose that the changing landscape of factors binding near Run over time can influence whether this factor functions to repress (“+R,” left) or activate (“+A,” right) expression of *sog_Distal*. See also Figures S2 and S3 and Videos S1, S2, S3, S4 and S5.

Using this quantitative analysis, we provided insights into dynamics of expression domain widths associated with *sog_Distal* variants over time. Specifically, we found that at nc13 the width of reporter expression is expanded relative to wild-type when Su(H) or Run sites are mutated or mutated in combination (see Figures 4A, 4B, 4D, and 4E, red numbers in top right of boxes). However, surprisingly, at later time points (i.e., nc14b,c), only the Su(H) mutant exhibited a clearly expanded expression domain (Figure 4B, compare with Figure 4A; red numbers in boxes in middle and right). Plots for representative embryos were directly overlaid to examine relative trends showing that *sog*_*D*_*_ΔSuH* exhibits a higher number of active nuclei and is likely dorsally as well as ventrally expanded, whereas lower active nuclei counts and widths closer to wild-type are associated with *sog*_*D*_*_Δrun* and the double mutant (Figure 4C).

To obtain more confidence in these trends, we conducted a small-scale statistical analysis that required we image four to seven embryos of each genotype and compare widths for reporter outputs at four time points: nc13, nc14a, nc14b, and nc14c. This small-scale statistical analysis (see Figure 4F) supports the view that Su(H) mutants exhibit outputs that are expanded in width at all time points, *sog*_*D*_*_Δrun* mutants only exhibit expansion early (nc13, nc14a) but not late (nc14b, nc14c), and the double mutant exhibits an intermediate phenotype: expanded early (nc13) but of normal width at nc14.

### The Temporally Changing Landscape of TFs Co-occupying Long-Acting Enhancers May Account for the Changing Roles of Inputs

Our data demonstrate that Run supports repression during early nuclear cycles but then switches to supporting activation at later stages. Evidence for Run switching can be obtained from careful analysis of staged, fixed embryo collections (Figures 4G, S3K, and S3L) but is more evident in the spatiotemporal gene expression dynamics captured by live imaging (Figures 3 and 4). This switch in Run activity likely relates to increasing influence from additional activators. For instance, in nc14, DLlevels progressively increase while also spatially refining to effect a more ventrally concentrated gradient (reviewed in Sandler and Stathopoulos, 2016); and studies have shown that Zld can also support activation of *sog_Distal* (Nien et al., 2011; Yamada et al., 2019). It is possible that DL/Zld and Run cooperate as activators to support expression of *sog_Distal* in late nc14. Furthermore, Run’s switch to supporting activation at nc14 can also explain why loss of Su(H) does not lead to an expanded gene expression output for the *sog_Distal* reporter in the context of the double *ΔSu(H)/run* mutant. The ability to assay loss of repression (i.e., expanded expression domain due to loss of the Su(H) repressor input) also requires the presence of required activator input (e.g., Run), to support expanded expression, in order that the phenotype be scorable.

Previous studies have focused on how Run and its vertebrate homolog Runx may act as repressor or activator on distinct enhancers, switching role depending on the TF landscape present; however, our data support an alternate mechanism. For instance, in the case of *Drosophila sloppy-paired 1* (*slp1*) gene regulation, Run activity is modulated in space depending on spatially localized TF co-occupancy to the DESE enhancer associated with the *slp1* locus (Hang and Gergen, 2017). Where the Fushi tarazu (Ftz) TF co-binds to the DESE enhancer, Run functions as a repressor; however, where Ftz is absent but the odd-paired (Opa) TF co-binds, Run functions as an activator. The human ortholog of Run, Runx, also interacts with several proteins that modulate its activity and support its different roles to regulate different enhancers (Chuang et al., 2013). Our data show for the first time that Run can also switch its activity in the context of regulating a single enhancer over time, and this ability may represent an additional general mechanism by which TFs contribute to the dynamics of gene expression outputs. Presumably, the TF binding landscape within a single enhancer also changes in time to temporally impact Run’s activity.

Both Run and Su(H)’s ability to influence spatiotemporal domains of gene expression (Figure 4H) may serve to counterbalance activators. In particular, we propose that broadly expressed or ubiquitous repressors are equally important as pioneer activators in managing the MZT. Future studies will aim to test the hypotheses that Run affects chromatin accessibility whereas Su(H) acts to impact RNA polymerase action. Importantly, this study demonstrates that broadly expressed or ubiquitous repressors have roles in regulating expression that differ from each other and yet both are likely pivotal to the MZT of early embryos.

## STAR★METHODS

### LEAD CONTACT AND MATERIALS AVAILABILITY

Further information and requests for resources and reagents should be directed to and will be fulfilled by the Lead Contact, Angelike Stathopoulos (angelike@caltech.edu).

### EXPERIMENTAL MODEL AND SUBJECT DETAILS

#### Fly Stocks and Husbandry

All flies were reared under standard conditions at 23°C, and *yw* background was used as wild-type unless otherwise noted.

#### Generation of Mutant Embryos

Embryos were depleted of maternal and zygotic Run by maternal expression of a short hairpin (*sh*) RNAi construct (Staller et al., 2013) directed to the *run* gene (i.e. *shRNA-run*). *UAS-shRNA-run* females [TRiP.HMS01186/TM3 - Bloomington *Drosophila* Stock Center (BDSC), stock #34707] were crossed to *MTD-Gal4* males (BDSC#31777). F1 *MTD-Gal4/UAS-shRNA-run* females were crossed back to *shRNA-run* males (#34707), and F2 embryos collected and assayed by *in situ* for *run* mutant phenotypes (Figure 4G). Prior to the analysis of the transgenic *run* RNAi, both *shRNA-run* expressing (i.e. *run* RNAi) and classical *run* mutant embryos were stained by *in situ* hybridization using a *sog* riboprobe, as described (Koromila and Stathopoulos, 2017). The *sog* expression patterns from both lines were compared and confirmed to be very similar to each other (data not shown). Both male and female embryos were examined; sex was not determined but assumed to be equally distributed.

#### Heat Shock-Mediated Ectopic Expression

For ectopic expression experiments, a transgenic line containing a heat-shock inducible *run* construct was used. For heat-shock experiments, 1–3 hr-old embryos carrying one copy of *hs-run* (Tsai and Gergen, 1994) and one copy of a given reporter gene were collected and transferred into a 37°C incubator for 20–25 min, allowed to recover at 25°C for 35–40 min, and fixed immediately (Koromila and Stathopoulos, 2017). Controls include comparisons of reporter expression associated with (i) embryos containing the *hs-run* construct without the heat-shock treatment as well as (ii) heat-shocked *yw* embryos lacking the construct (Figure S3,K,L). Both male and female embryos were examined; sex was not determined but assumed to be equally distributed.

### METHOD DETAILS

#### Cloning and Transgenic Fly Construction

Construction of *sog_*Intronic.*lacZ* construct was previously described (Koromila and Stathopoulos, 2017; Liberman and Stathopoulos, 2009). *sog_Distal* sequences with mutated Su(H)/Run-binding sites [i.e., *sog*_*D*_*_ΔSu(H), sog*_*D*_*_Δrun, sog*_*D*_*_ΔSu(H)/run* and *sog*_*D*_*_add_run*] were chemically synthesized (GenScript) and ligated into the *eve2 promoter-MS2.yellow*-attB vector (Bothma et al., 2014) using standard cloning methods, as previously described (Koromila and Stathopoulos, 2017). Site-directed transgenesis of these reporters was carried out using a *D. melanogaster* stock containing attP insertion site at position ZH-86Fb (Bloomington *Drosophila* Stock Center #23648) (Bischof et al., 2007).

Sequence of the *sog*_Distal enhancer (658 bp) and position of Su(H) and Run binding sites, in bold, are shown below:

GCGGCCGCGACAGATTCCCGGGTTTCAGCGGAACAGGTAGGCTGGTCGATCGGAAAT**TCCCACC**ATACACATGTGGCTATAATGCCAACGGCATCGAGGTGCGAAAACAGATGCAGCCTCATAAAAGGGGCGCAGATAAGGTCGCGGTTGC**GTGGGAA**AAGCCCATCCGACCAGGACCAGGACGAAGCAG**TGCGGTT**GGCGCATCATTGCCGCCATATCTGCTATTCCTACCTGCGTGGCCATGGCGATATCCTTGTGCAAGGATAAGGAGCGGGGATCATAAAACGCTGTCGCTTTTGTTTATGCTGCTTATTTAAATTGGCTTCTTGGCGGGCGTTGCAACCTGGTGCTAGTCCCAATCCCAATCCCAATTCCAATCCGTATACCCGTATATCCAATGCATTCTACCTGTCCTGGGAATTTCCGATTTGGCCGCACCCATATGGCCACGGATGC**GTGAGAG**TGCTCTCCGTGCGATTCTAGATCATCGTGGGTATTCGCAGACAATCGGGTTATTGTGCCGCATTCGATGTTGGCTCTTTGGTTTTCGGAAACTCTGACCAGGTTTTCGGTTTTCGGTTTTTGATTTTGGGTTTTTCCGGCCGCATCGTGCGTCATCTGGTGGCACAGGACGCACTTGCCCCTGTCAGTTAGATCT

Mutated site sequences (capitalized text) and corresponding wild-type sequences (lowercase text) are as follows:

- *sog*_*D*_*_ΔSu(H)*: ttcccacc > ttcccGA-, gtgggaa > ACAAgaa, gtgagag > ACAagag
- *sog*_*D*_*_Δrun*: tgcggtt > tAcgAtt
- *sog*_*D*_*_ΔSu(H)/run*: ttcccacc > ttcccGA-, gtgggaa > ACAAgaaa, gtgcggtt > gtAcgAtt, gtgagag > ACAagag
- *sogD_add_run:*cgcggtt > Tgcggtt

Based on the current literature, the single Runt binding site found in *sog*_Distal enhancer sequence is a perfect match to the DNA-binding consensus sequence defined for vertebrate ortholog Runx1 and a perfect match to Run binding sites analyzed previously in *Drosophila* (Melnikova et al., 1993). The PWM used (i.e., ACCRCA) relates to binding of the vertebrate homolog Runx (Lewis et al., 1999), but *Drosophila* studies have used it to guide analysis of *Drosophila* Runt binding sites using a slightly extended PWM of AACCRCA (e.g., Chen et al., 2012). Specifically, the *sog_Distal* enhancer contains a perfect match to the consensus sequence that was mutated in the context of *sogD_Δrun* (i.e., AACCGCA). The next best site present in *sog*_Distal enhancer has a mismatch within the core consensus (i.e., AACCGCg) and is not likely to bind Runt. However, mutagenesis of this other site was performed in order to transform it into a perfect match to the consensus binding site by introducing a one basepair change (g > A) in the context of construct *sogD_add_run*.

The three Su(H) binding sites within *sog_Distal* enhancer (i.e., RTGRGAR) were identified previously in the context of a *sog_DistallacZ* reporter analysis (Ozdemir et al., 2014) and mutated for the purposes of this study as described above.

#### *In situ* Hybridizations and Image Processing

Embryos were collected, fixed, and stained using intronic *yellow* and *sog* riboprobes labeled with digoxigenin (DIG) using standard conditions. To assay reporter expression in *sog*_*D*_*_Δrun* at nc9, embryos were fluorescently stained with DIG-labeled intronic *yellow* riboprobes DAPI to visualize nuclei. Imaging was performed with a Zeiss Axioxam 506 microscope using a 20x objective.

### QUANTIFICATION AND STATISTICAL ANALYSIS

#### Live Imaging Optimization and Data Acquisition

For quantitative real-time imaging, we used the MS2.MCP RNA stem loop-based system and all analysis was carried out using MATLAB R2017b. In order to monitor the various *sog_Distal* reporters described above in live embryos, female virgins of line *yw;Nucleoporin-RFP;MCP-NoNLS-GFP* were crossed with MS2 reporter-containing males. Embryos were collected one hour after egg laying, dechorionated by hand not using bleach (an important modification to other protocols), mounted between a glass slide and a coverslip using heptane-dissolved glue with folded double-sided tape used between the slide and the coverslip to allow a limited amount of embryo flattening, and embedded in Halocarbon 27 oil (Sigma-Aldrich). The flattening of the embryos makes it possible to image more nuclei in the same focal plane without causing any detectable change to early developmental processes.

Embryos were imaged on a Zeiss LSM 800 confocal microscope using a 25x oil immersion objective. To increase time-resolution, we used a 0.7 digital magnification without limiting the scanned field of the entire embryo. The MCP-GFP and Nucleoporin-RFP were excited with laser wavelengths of 488nm and 561nm, respectively. Images were captured at 512 × 512 pixel resolution with the pinhole set to a diameter of 50 μm. At each time point, a stack of 26–30 z-plane images separated by 0.55 μm were captured, spanning the nuclear layer. The final time interval was 65–75 s, and imaging sessions were maximally two hours in length. To capture expression from nc9-nc14c required imaging two different embryos (i.e., Videos S1A, S2A, S3A, and S5B), because this developmental window encompasses 3_+_ hours.

The number of embryos imaged per nuclear cycle per constructs *sog_Distal, sogD_Δrun, sogD_ΔSu(H), and sogD_ΔSu(H)/run* are as follows:

- Early movies (Videos S1A, S2A, S3A, and S4A; total 3 embryos/construct); nc9, nc10, nc11, nc12, nc13, nc14a and nc14b; average n of frames for wt, *Δrun, ΔSu(H), ΔSu(H)/run*: 61, 63, 65, 64, respectively.
- Late movies (Video S5B wt; check below for total number of embryos/construct/nc): nc13, nc14a-nc14c; average n of frames for wt, *Δrun, ΔSu(H), ΔSu(H)/run*: 45, 50, 47, 32, respectively:
  nc13; 1 embryo/construct (but also covered by early movies, see above)
  nc14a-nc14c; average of 3 embryos/construct.
  Three embryos were imaged for construct *sogD_add_run* in movies that span nc13–14c. Average n of frames is 31.

#### Segmentation

Segmentation of Nucleoporin-RFP (Nup-RFP) nuclei and detection of MCP-GFP+ dots associated with MS2-containing nascent transcript were performed on the movies with MATLAB using customized scripts. To start analysis of data for nc11 or later time points, MCP-GFP and Nup-RFP slices were maximally projected for each time point using Fiji software.

For segmentation of the signal in both channels, we first used a Gaussian Filter (GF) (GF = 0.55) in Fiji software as used in other MS2-MCP *Drosophila* imaging pipelines (Bothma et al., 2014; Garcia et al., 2013), to both reduce pixel-level noise and to smooth small-scale image variations within a single object. Next, we developed a MATLAB computational pipeline to process images from the two channels (488nm channel: MCP-GFP, 555nm channel: Nup-RFP) used to collect our data, which consisted of approximately 2 hours of imaging and ~80 scans. Specifically: (I) Embryo boundaries were detected in 3D using Nup-RFP filtered images, using customized code. We delineated the embryo boundary in a semi-automated fashion by manually defining extreme dorsal and ventral points. The boundary coordinates were then propagated along the MCP-GFP iso-intensity lines using MATLAB’s *bwboundary* function. (II) Nascent dot intensity determination requires an estimate of the background for each dot for the *sog_Distal.MS2* transgene. We used scripts for segmentation of both MCP-GFP and Nup-RFP signals. MCP-GFP labeled transcriptional dots falling outside Nup-RFP labeled nuclei were excluded. To segment the transcriptional dots in a uniform manner across all datasets, we applied the Gaussian Filter (GF) using Fiji Software as well as thresholded the MCP-GFP channel at 30% (i.e., BTH = 0.30) of maximum intensity (e.g., Figures 3F, 3H, and S2D; Background Threshold (BTH): gray dotted line). A less stringent threshold point (BTH = 0.25) was tested but rejected (data available for comparison Figures S3A, S3B, and S3E) because false positives were obtained for early time point at nc9. Only the 30% threshold (BTH = 0.3) eliminates the background from the non-nuclear regions in a conservative manner for all constructs assayed. BTH = 0.35 was explored but resulted in over 40% loss of signal at many time points, and therefore was only used for comparison sake (Figures S3C, S3D, and S3F).

Finally, after thresholding and before quantification analysis, the segmentation and tracking was also visually checked frame by frame over multiple nuclear cycles in order to verify the dot’s location relative to nuclear membranes (Nup-RFP). An analysis of these measurements for three to six embryos was performed to define the number of total active nuclei and associated standard error of the mean.

#### Image Analysis and Data Acquisition

Segmented dots were defined as clusters of adjacent (4-connected) pixels above the threshold. Black traces represent counts of active nuclei/dots of MS2-MCP signal detected throughout embryos (Figures 4A, 4B, 4D, and 4E). Finally, ventral boundary position (*sogD_VB*) is defined as the ventral-most position at which signal-traces decrease to a level that passes below threshold (BTH), representing where the Snail (or other) repressor would normally act to repress *sog* expression [Figure S2D, horizontal gray dashed line “(2)”]. Initiation time for the transcriptional activity of the *sog_Distal.MS2* in each nucleus is defined as the time corresponding to the first frame at which a dot was detected.

Imaris Bitplane software was used to identify the thresholded data only for presentation purpose (replaced GFP_+_ dots with blue small sphere, Figures 2D, 3A–3E, and S2C) but did not relate to quantification, which instead relied solely on processing of the background corrected, raw intensity signals.

For each of the four enhancer constructs *sog*_*D*,_ *sog*_*D*_*_ΔSu(H), sog*_*D*_*_Δrun*, and *sog*_*D*_*_ΔSu(H)/run*, the number of active nuclei was calculated by averaging the MS2-MCP dots from all the frames per ~10 min time window representing particular developmental stage (i.e., nc9, 10, 11, 12, 13, 14a, 14b, or 14c) per embryo (Figure 3F).± 2°C difference in the microscope’s room temperature might cause small variations in the number of frames per stage due to inherent variability in timing of early development. Therefore, the number of frames was not equivalent for any particular nuclear cycle time point. Either one representative scan from each nuclear cycle or an average of all scans within each nuclear cycle was assayed, as noted.

#### Average Dot Size Calculation

The rate of transcription was also calculated based on the average dots’ size per time window, defined by the MS2 x MCP-GFP pixels/dot ratio (Figure 3H), as described above. For this particular analysis, the size of each dot was calculated as the average number of pixels per dot, which is itself defined as having 4-adjacent pixels, and presented as an average pixel to dot ratio for each of the specified time periods associated with each of the four constructs. Note that as each dot is called only if 4-adjacent pixels are present, the absolute number of pixels is 4 times the average dot size. Furthermore, for each time point, dot sizes were calculated for each of the individual frames present per time window (i.e., nc13, nc14a, nc14b or nc14c) and averaged, for each embryo. Such data were obtained for four embryos at nc13 and six for the other time points per construct, averaged, and this number plotted. Significance of replicates was tested using a Student’s two-tailed t test and significance designated by a p value of < 0.05 and noted by asterisk in Figure 3H when different from the wt enhancer.

We note that detection of transcription at early time points (nc9 to nc12) or earlier is challenging to visualize since nuclei have not yet completed migration to the periphery and therefore are located deep in the embryo. Therefore, to analyze expression at such early time points, individual confocal stacks, not projections, of *sog*_*D*,_ *sog*_*D*_*_ΔSu(H), sog*_*D*_*_Δrun*, and *sog*_*D*_*_ΔSu(H)/run* constructs (three embryos per construct) were used for transcriptional activity estimation during nuclear cycles 9.

#### Width Quantification of Dynamic sog_Distal Output

For quantification of width of expression along the DV axis, we quantified the relative size of expression patterns along the Embryo Width (EW) axis in order to make comparisons between embryos. In order to reduce data dimensionality in the following analysis, only the dorsoventral axis (i.e., EW) component of nascent dot position was taken into consideration, ignoring the AP coordinates. To visualize expression dynamics, we plot a histogram over the EW axis using bins of 4 pixels. Next, expression domain dynamics were quantified using Kernel Density Estimation (KDE) (Silverman, 1986). KDE fits a probability density curve to the spatial distribution of active nuclei relative to EW axis position (smoothing curve; Figures 4A, 4B, 4D, and 4E). The pixel-cluster’s geometric center was used to define the dot’s location as a single-pixel coordinate in order to avoid assigning a single dot (composed of a contiguous set of adjacent pixels) to multiple bins. This was done independently for each time point (i.e., scan) for all the nuclear cycles imaged during the course of the 2-hour movie. A mixture of Gaussian kernels, with kernel bandwidth estimated using “Silverman’s rule,” was used which assumes a near-normal unimodal distribution (Silverman, 1986).

To determine a value representing expression domain width that could be used to compare data for different time points or embryos, we used an empirically defined, predetermined fraction of the KDE curve maximum. For the measurement of *sog_Distal* enhancer output width, we tested three different max fractions, or thresholds: TH = 0.25, TH = 0.3 and TH = 0.35. In our case, the embryo-to-embryo variability was found to be minimized at threshold of 30% of the maximum observed signal (TH = 0.3). Minimization of variability was necessary to allow detection of differences between populations of wt and mutant embryos. An analysis of these measurements for four to six embryos was performed to define the average width measurements (length of dashed red line in EW relative units; e.g., see Figures 4A, 4B, 4D, and 4E) and associated standard error of the mean (Figure 4F). We compared the width of the reporters’ expression domain at all four time points (i.e., nc13, nc14a, nc14b and nc14c).

### DATA AND CODE AVAILABILITY

The code generated during this study is available at GitHub (see Key Sources Table).

## Supplementary Material

1

2

3

4

5

6

7

## Figures and Tables

**Table:** KEY RESOURCES TABLE

REAGENT or RESOURCE	SOURCE	IDENTIFIER
Chemicals, Peptides, and Recombinant Proteins		
DAPI	Invitrogen	D3571
Digoxigenin labeled nucleotides	Roche	11277073910
Phusion DNA polymerase	NEB	M0530S
Halocarbon 27 oil	Sigma-Aldrich	MKBJ5699
Experimental Models: Organisms/Strains		
*D. melanogaster.ZH-86Fb*	Bloomington Drosophila Stock Center (BDSC)	23648
D. melanogaster.*sog*_lntronic.*lacZ*	Koromila and Stathopoulos, 2017	TK38
D. melanogaster.*sog*_*Distal*	This study	TK54
D. melanogaster.*sog*_*D*__*ΔSu(H)*	This study	TK55
D. melanogaster.*sog*_*D*__*Δrun*	This study	TK56
D. melanogaster.*sog*_*D*__*ΔSu(H)*/*run*	This study	TK57
D. melanogaster.*sog*_*D*__*add*_*run*	This study	TK58
D. melanogaster*.yw;Nucleoporin-RFP;MCP-NoNLS-GFP*	Lucas et al., 2013	TK59
D. melanogaster.*hs*-*run*	Tsai and Gergen, 1994	TK60
D. melanogaster.*UAS-shRNA-run*	BDSC	34707
D. melanogaster.*MTD*-*Gal4*	BDSC	31777
Recombinant DNA		
*eve2 promoter*-*MS2.yellow*-attB	Bothma et al., 2014	N/A
*sog_Distal eve2 promoter*-*MS2.yellow-*attB	This study	TK54_DNA
*sog*_*D*_*_Δrun eve2 promoter*-*MS2.yellow-*attB	This study	TK55_DNA
*sog*_*D*_*_ΔSu(H) eve2 promoter*-*MS2.yellow-*attB	This study	TK56_DNA
*sog*_*D*_*_ΔSu(H)/run eve2 promoter*-*MS2.yellow-*attB	This study	TK57_DNA
*sog*_*D*_*_add_run eve2 promoter*-*MS2.yellow-*attB	This study	TK58_DNA
Other		
*sog_Distal* sequences with mutated Su(H)/Run-binding sites were chemically synthesized	GenScript	N/A
Software and Algorithms		
JASPAR	Khan et al., 2018	http://jaspar.binf.ku.dk/cgi-bin/jaspar_db.pl?rm=browse&db=core&tax_group=insects
Fiji	Schindelin et al., 2012	N/A
Imaris 9.0		N/A
Kernel Density Estimation (KDE) algorithm	Silverman, 1986	N/A
GitHub	This study	https://github.com/tkoromila/ldentity_Crisis
Other		
*sog_Distal* sequences with mutated Su(H)/Run-binding sites were chemically synthesized	GenScript	N/A

## Highlights

- Roles for broadly expressed repressors, Su(H) and Runt, investigated by live imaging
- Mutant and wild-type sog_Distal enhancer reporter outputs were quantitatively compared
- Su(H) impacts transcription levels whereas Run regulates transcription initiation
- Run switches roles in time from repressor to activator

## References

[R1] Bertrand E, Chartrand P, Schaefer M, Shenoy SM, Singer RH, and Long RM (1998). Localization of ASH1 mRNA particles in living yeast. Mol. Cell 2, 437–445. 9809065 10.1016/s1097-2765(00)80143-4

[R2] Bischof J, Maeda RK, Hediger M, Karch F, and Basler K (2007). An optimized transgenesis system for Drosophila using germ-line-specific phiC31 integrases. Proc. Natl. Acad. Sci. USA 104, 3312–3317. 17360644 10.1073/pnas.0611511104 PMC1805588

[R3] Bothma JP, Garcia HG, Esposito E, Schlissel G, Gregor T, and Levine M (2014). Dynamic regulation of eve stripe 2 expression reveals transcriptional bursts in living Drosophila embryos. Proc. Natl. Acad. Sci. USA 111, 10598–10603. 24994903 10.1073/pnas.1410022111 PMC4115566

[R4] Chen H, Xu Z, Mei C, Yu D, and Small S (2012). A system of repressor gradients spatially organizes the boundaries of Bicoid-dependent target genes. Cell 149, 618–629. 22541432 10.1016/j.cell.2012.03.018 PMC3535481

[R5] Chuang LSH, Ito K, and Ito Y (2013). RUNX family: Regulation and diversification of roles through interacting proteins. Int. J. Cancer 132, 1260–1271. 23180629 10.1002/ijc.27964

[R6] Dunipace L, Saunders A, Ashe HL, and Stathopoulos A (2013). Autoregulatory feedback controls sequential action of cis-regulatory modules at the brinker locus. Dev. Cell 26, 536–543. 24044892 10.1016/j.devcel.2013.08.010 PMC3782659

[R7] Ferraro T, Esposito E, Mancini L, Ng S, Lucas T, Coppey M, Dostatni N, Walczak AM, Levine M, and Lagha M (2016). Transcriptional Memory in the Drosophila Embryo. Curr. Biol 26, 212–218. 26748851 10.1016/j.cub.2015.11.058 PMC4970865

[R8] Foo SM, Sun Y, Lim B, Ziukaite R, O’Brien K, Nien C-Y, Kirov N, Shvartsman SY, and Rushlow CA (2014). Zelda potentiates morphogen activity by increasing chromatin accessibility. Curr. Biol 24, 1341–1346. 24909324 10.1016/j.cub.2014.04.032 PMC4075064

[R9] Garcia HG, Tikhonov M, Lin A, and Gregor T (2013). Quantitative imaging of transcription in living Drosophila embryos links polymerase activity to patterning. Curr. Biol 23, 2140–2145. 24139738 10.1016/j.cub.2013.08.054 PMC3828032

[R10] Hang S, and Gergen JP (2017). Different modes of enhancer-specific regulation by Runt and Even-skipped during *Drosophila* segmentation. Mol. Biol. Cell 28, 681–691. 28077616 10.1091/mbc.E16-09-0630 PMC5328626

[R11] Harrison MM, Li X-Y, Kaplan T, Botchan MR, and Eisen MB (2011). Zelda binding in the early Drosophila melanogaster embryo marks regions subsequently activated at the maternal-to-zygotic transition. PLoS Genet. 7, e1002266. 22028662 10.1371/journal.pgen.1002266 PMC3197655

[R12] Hong J-W, Hendrix DA, and Levine MS (2008). Shadow enhancers as a source of evolutionary novelty. Science 321, 1314. 18772429 10.1126/science.1160631 PMC4257485

[R13] Ip YT, and Hemavathy K (1997). Drosophila development. Delimiting patterns by repression. Curr. Biol 7, R216–R218. 9162494 10.1016/s0960-9822(06)00104-7

[R14] Khan A, Fornes O, Stigliani A, Gheorghe M, Castro-Mondragon JA, van der Lee R, Bessy A, Chèneby J, Kulkarni SR, Tan G, (2018). JASPAR 2018: update of the open-access database of transcription factor binding profiles and its web framework. Nucleic Acids Res 46 (D1), D1284. 29161433 10.1093/nar/gkx1188 PMC5753202

[R15] Koromila T, and Stathopoulos A (2017). Broadly expressed repressors integrate patterning across orthogonal axes in embryos. Proc. Natl. Acad. Sci. USA 114, 8295–8300. 28720706 10.1073/pnas.1703001114 PMC5547611

[R16] Laver JD, Marsolais AJ, Smibert CA, and Lipshitz HD (2015). Regulation and Function of Maternal Gene Products During the Maternal-to-Zygotic Transition in Drosophila. Curr. Top. Dev. Biol 113, 43–84. 26358870 10.1016/bs.ctdb.2015.06.007

[R17] Lewis AF, Stacy T, Green WR, Taddesse-Heath L, Hartley JW, and Speck NA (1999). Core-binding factor influences the disease specificity of Moloney murine leukemia virus. J. Virol 73, 5535–5547. 10364302 10.1128/jvi.73.7.5535-5547.1999 PMC112611

[R18] Liberman LM, and Stathopoulos A (2009). Design flexibility in cis-regulatory control of gene expression: synthetic and comparative evidence. Dev. Biol 327, 578–589. 19135437 10.1016/j.ydbio.2008.12.020 PMC2746413

[R19] Long HK, Prescott SL, and Wysocka J (2016). Ever-Changing Landscapes: Transcriptional Enhancers in Development and Evolution. Cell 167, 1170–1187. 27863239 10.1016/j.cell.2016.09.018 PMC5123704

[R20] Lucas T, Ferraro T, Roelens B, De Las Heras Chanes J, Walczak AM, Coppey M, and Dostatni N, (2013). Live imaging of bicoid-dependent transcription in Drosophila embryos. Curr. Biol 23, 2135–2139. 24139736 10.1016/j.cub.2013.08.053

[R21] MacArthur S, Li X-Y, Li J, Brown JB, Chu HC, Zeng L, Grondona BP, Hechmer A, Simirenko L, Keränen SVE, (2009). Developmental roles of 21 Drosophila transcription factors are determined by quantitative differences in binding to an overlapping set of thousands of genomic regions. Genome Biol 10, R80. 19627575 10.1186/gb-2009-10-7-r80 PMC2728534

[R22] Melnikova IN, Crute BE, Wang S, and Speck NA (1993). Sequence specificity of the core-binding factor. J. Virol 67, 2408–2411. 8445737 10.1128/jvi.67.4.2408-2411.1993 PMC240414

[R23] Nien C-Y, Liang H-L, Butcher S, Sun Y, Fu S, Gocha T, Kirov N, Manak JR, and Rushlow C (2011). Temporal coordination of gene networks by Zelda in the early Drosophila embryo. PLoS Genet. 7, e1002339. 22028675 10.1371/journal.pgen.1002339 PMC3197689

[R24] Ozdemir A, Ma L, White KP, and Stathopoulos A (2014). Su(H)-mediated repression positions gene boundaries along the dorsal-ventral axis of Drosophila embryos. Dev. Cell 31, 100–113. 25313963 10.1016/j.devcel.2014.08.005 PMC4201238

[R25] Perry MW, Bothma JP, Luu RD, and Levine M (2012). Precision of hunchback expression in the Drosophila embryo. Curr. Biol 22, 2247–2252. 23122844 10.1016/j.cub.2012.09.051 PMC4257490

[R26] Sandler JE, and Stathopoulos A (2016). Stepwise Progression of Embryonic Patterning. Trends Genet. 32, 432–443. 27230753 10.1016/j.tig.2016.04.004 PMC5065017

[R27] Schindelin J, Arganda-Carreras I, Frise E, Kaynig V, Longair M, Pietzsch T, Preibisch S, Rueden C, Saalfeld S, and Schmid B (2012). Fiji: an open-source platform for biological-image analysis. Nat Methods. 9, 676–682. 22743772 10.1038/nmeth.2019 PMC3855844

[R28] Silverman BW (1986). Density Estimation for Statistics and Data Analysis (Chapman and Hall).

[R29] Staller MV, Yan D, Randklev S, Bragdon MD, Wunderlich ZB, Tao R, Perkins LA, Depace AH, and Perrimon N (2013). Depleting gene activities in early Drosophila embryos with the “maternal-Gal4-shRNA” system. Genetics 193, 51–61. 23105012 10.1534/genetics.112.144915 PMC3527254

[R30] Stathopoulos A, and Levine M (2005). Genomic regulatory networks and animal development. Dev. Cell 9, 449–462. 16198288 10.1016/j.devcel.2005.09.005

[R31] Tsai C, and Gergen JP (1994). Gap gene properties of the pair-rule gene runt during Drosophila segmentation. Development 120, 1671–1683. 8050373 10.1242/dev.120.6.1671

[R32] Yamada S, Whitney PH, Huang S-K, Eck EC, Garcia HG, and Rushlow CA (2019). The Drosophila Pioneer Factor Zelda Modulates the Nuclear Microenvironment of a Dorsal Target Enhancer to Potentiate Transcriptional Output. Curr. Biol 29, 1387–1393.e5. 30982648 10.1016/j.cub.2019.03.019 PMC6702943

[R33] Yuh CH, and Davidson EH (1996). Modular cis-regulatory organization of Endo16, a gut-specific gene of the sea urchin embryo. Development 122, 1069–1082. 8620834 10.1242/dev.122.4.1069

