# Supplementary material for: Distinct Roles of Broadly Expressed Repressors Support Dynamic Enhancer Action and Change in Time
Source: Cell Rep. Author manuscript; Available in PMC 2019 Dec 23. (PMC6927530; doi:10.1016/j.celrep.2019.06.063)
Supplement: 1 [file NIHMS1535567-supplement-1.pdf]

Cell Reports, Volume *28*

Supplemental Information

Distinct Roles of Broadly Expressed Repressors Support Dynamic Enhancer Action and Change in Time

Theodora Koromila and Angelike Stathopoulos

SUPPLEMENTAL FIGURES AND FIGURE LEGENDS

Supplemental_ Fig_S1

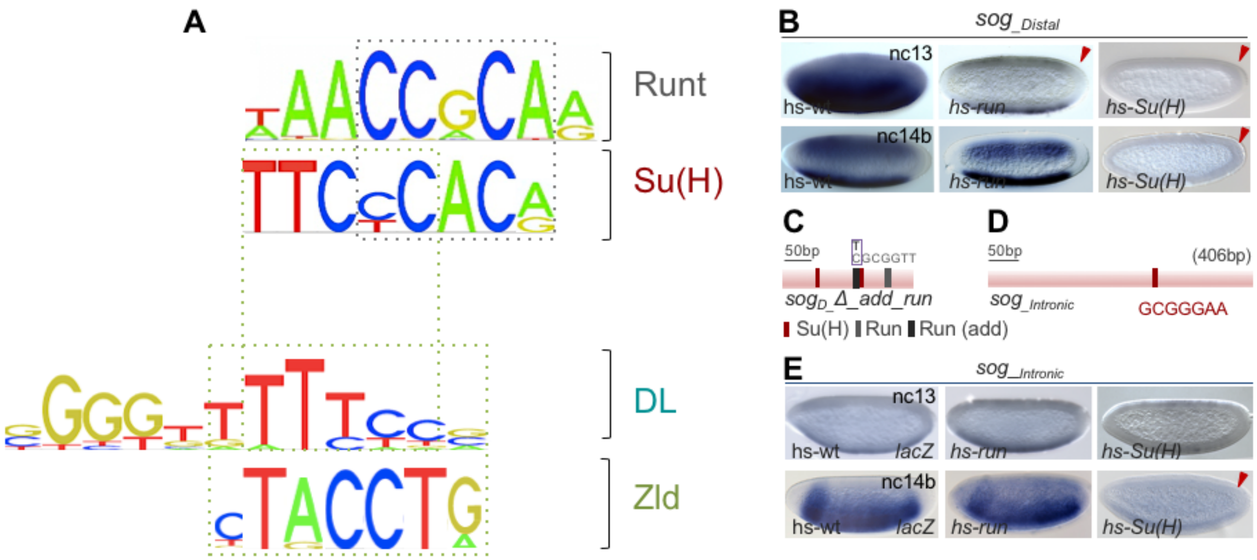

Supplemental_Fig_S1. Presence of binding sites in *sog* early embryonic enhancers correlates with ability of broadly-expressed repressors to mediate repression. Relates to Fig. 1.

(A) Consensus binding sites for transcription factors, Run, Su(H), DL, and Zld, aligned to demonstrate similarities in the binding sequences using JASPAR (Khan et al., 2018) **(B)** Heat-shocked embryos overexpressing *run* or *Su(H)* via *hs-run* or *hs-Su(H)* constructs results in changes to *sog_Distal LacZ* reporter outputs. Embryos were stained by *in situ* hybridization using a *LacZ* riboprobe. *hs*-wt refers to *yw* control embryos containing the reporter that were equivalently processed. **(C)** Diagram of section of *sog_Distal* enhancer region showing location of additional Run site introduced upon mutagenesis (black) relative to original Run (gray) and Su(H) (red) binding sites . **(D)** Diagram of the *sog_Intronic* enhancer depicting the single match to Su(H) binding site consensus (red), and absence of Run binding site consensus match. **(E)** Ectopic expression of Run or Su(H) (see B) exhibits assayable effect on *sog_Intronic LacZ* reporter output only at nc14b, and only for Su(H).

Supplemental_ Fig_S2

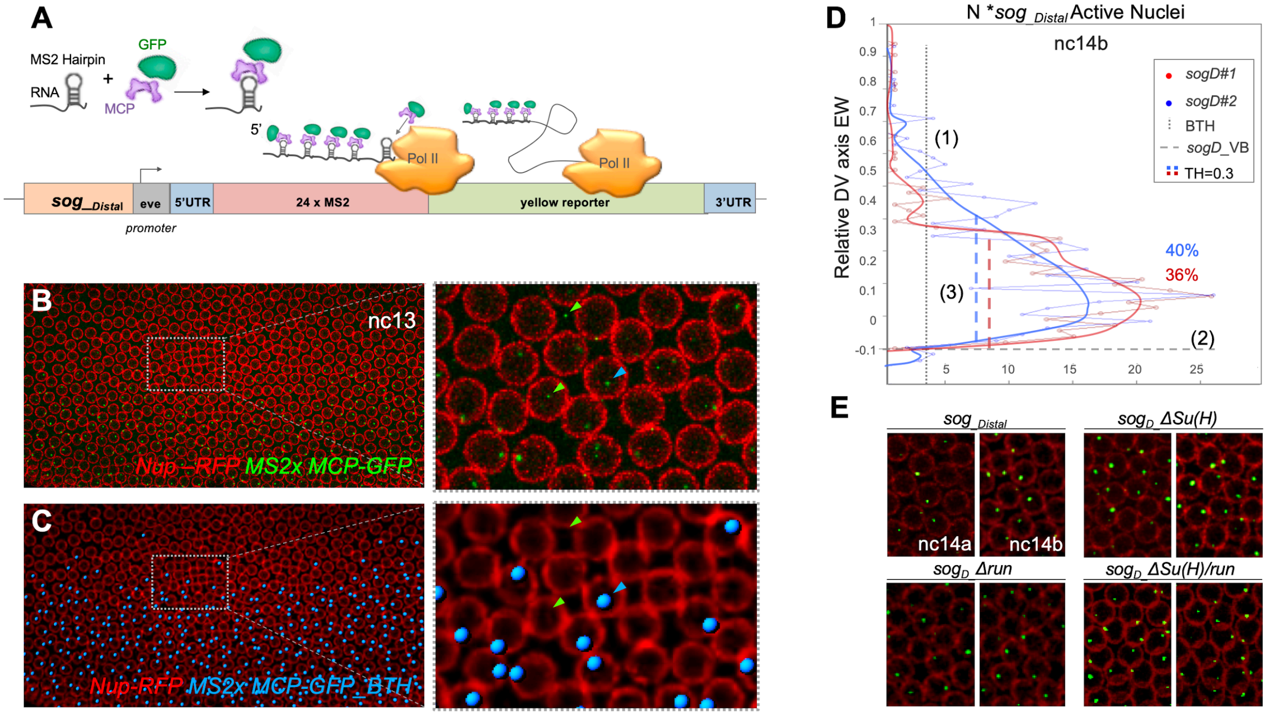

Supplemental_ Fig_S2. MS2-MCP system supporting live imaging of *sog_Distal* reporter gene expression, and background thresholding and quantification parameters used to generate width measurements of patterns along the DV axis. Relates to Figs. 2, 3, and 4. **(A)** Schematic showing components of the MS2-MCP system (Bertrand et al., 1998) that supports the *in vivo* imaging approach used. *sog_Distal* wildtype or mutant enhancer sequences were individually cloned upstream of a heterologous promoter driving a two-component reporter gene containing both 24 copies of the MS2 sequence, which encode RNA hairpins bound by MCP-GFP, and the *Drosophila yellow* gene with native introns using a previously published vector (see Materials & Methods; Bothma et al., 2014). **(B,C)** Raw MS2 x MCP-GFP imaging data (green; B) was computationally filtered to eliminate background (see Materials & Methods) and then processed using Imaris Bitplane software for visualization purposes in which green dots were replaced by small green spheres (blue; C). Dashed boxes represents the area magnified and shown

to the right. Green arrowheads in magnified views indicate dots that did not meet the threshold criteria for quantification (i.e. see BTH, below, see D) and were therefore excluded from analysis. The blue arrowhead identifies a representative dot in the raw data (B) that passed both filtering and thresholding (i.e. both dot's size was large enough and localization was confirmed as inside the nucleus), and thus remains present after processing (C). **(D)** Shown is a graph of signal across the entire lateral side of two *sog_Distal* embryos (embryo 1: blue and embryo 2: red) for either raw MS2-MCP signal (raw datapoints collapsed along the AP axis plotted in terms of EW position to mark relative DV axis position) or after smoothing curve correction (solid lines). To quantify widths in this imaging data, the following systematic filtering protocol was used to process data for each embryo: (1) Gaussian Filter (GF) using Fiji software as well as optimal threshold was applied such that 30% of the total number of active nuclei were removed from consideration to conservatively eliminate background (BTH, vertical grey dotted line). (2) Ventral boundary position (*sogD_VB*) is defined as the most ventral position that intersects with background threshold (horizontal grey dashed line). (3) For the measurement of *sog_Distal*'s expression, we report width at 30% (TH=0.3) of the total number of active nuclei from the smoothing curve (i.e. KDE, TH=0.3; see Materials & Methods). In these examples, width was calculated as 40% in units of relative EW for embryo 1, whereas width was calculated as 36% for embryo 2 (see vertical blue and red dashed lines, respectively). **(E)** Magnified view of indicated reporter constructs does not show difference in size of nascent GFP positive dots associated with MS2-MCP signal within nuclei (Nup-RFP marked) at stages nc14a and nc14b.

Supplemental_ Fig_S3

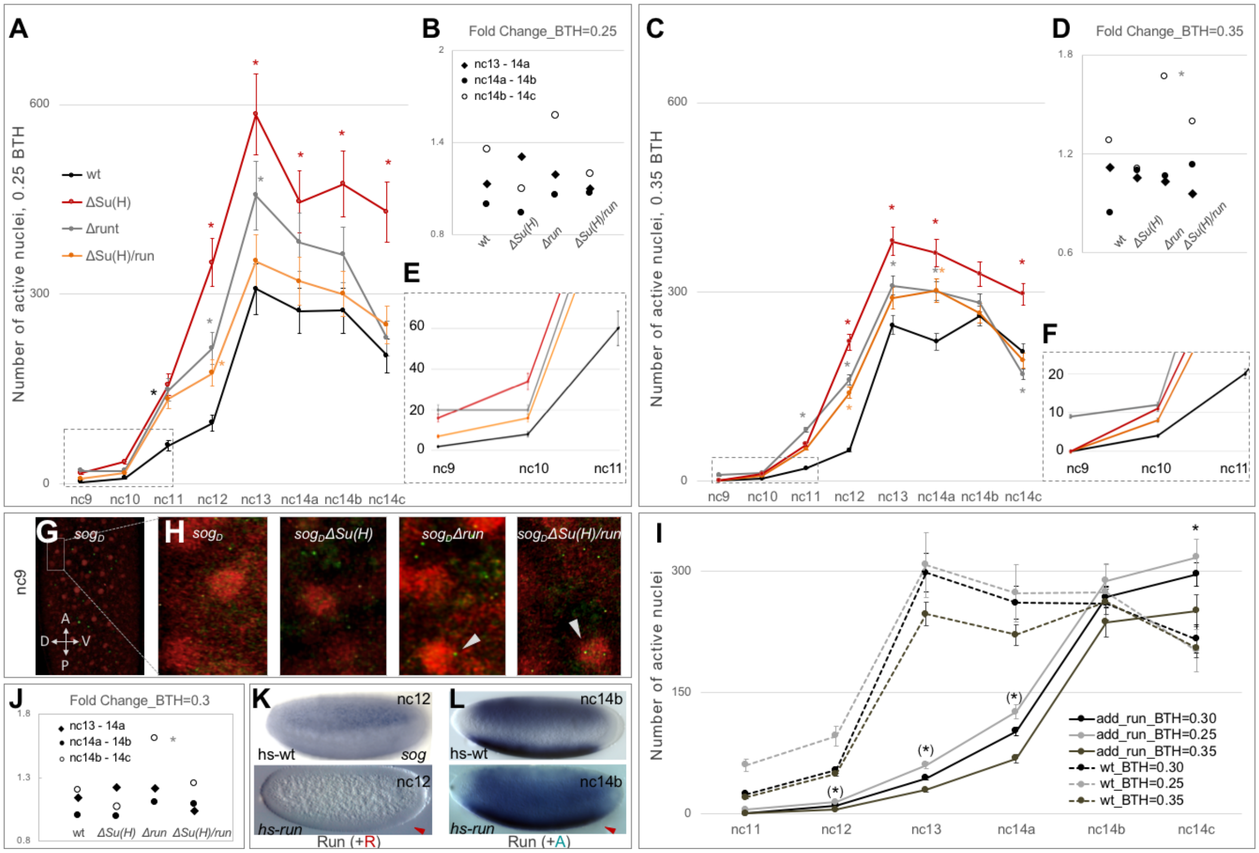

Supplemental_ Fig_S3. Data supporting threshold setpoints and gain-of-function *run* mutant phenotypes relating to *sog* gene expression. Relates to Figs. 3 and 4.

(A,C) Total active nuclei counts, MS2-MCP signal after background thresholding using setpoints of BTH=0.25 (A) or BTH=0.35 (C), averaged for all stills per embryo per timepoint from sets of movies spanning nc9 through nc14c. Such data were obtained and averaged for three to six representative movies of wild type or mutant variant *sog_Distal MS2-yellow* reporter constructs (see key, and Star Methods for details). Error bars represent SEM. **(B,D,J)** Fold change in total active nuclei counts per embryo averaged per timepoint pairs indicated spanning nc13 through nc14c using different thresholds: BTH=0.25 (B), BTH=0.3 (see Fig. 3F), and BTH=0.35 (D). **(E,F)** Magnified view of plots in (A) and (B)**,** respectively, with focus on nc9-11 data showing that in

all four indicated constructs active nuclei were detected as early as nc9 when a relaxed BTH=0.25 setpoint is used, which is not consistent with wildtype *sog_Distal* published information (Nien et al., 2011). **(G,H)** Stills of raw MS2xMCP-GFP imaging data from *sog_Distal* embryo at nc9. Box within the embryo or (G) represent the position of magnified views shown for wildtype or mutant constructs as labelled (H). The white arrowhead points to a nascent GFP positive dot within the nucleus in $sog_D\_\Delta run$ and $sog_D\_\Delta Su(H)/run$ at nc9; other constructs do not show expression. **(I)** Total number of active nuclei after different background thresholding (BTH=0.25/0.3/0.35, as indicated), per embryo, averaged for all stills per timepoint, spanning nc11 through nc14c. Moreover, such data were obtained and averaged for two (nc11-12) and three (nc13, 14a,b,c) representative movies of wildtype or mutant variant *sog_Distal_add_run* MS2-yellow reporter construct. The error bars represent SEM. Asterisks refer to statistically significant comparisons versus control (i.e. wt, dotted lines as indicated for each BTH) at BTH=0.30 at the same timepoint. Asterisks in parenthesis refer to inverse correlation with wt. **(K,L)** Gain-of-function (heat shock construct *hs-run*) *run* mutant embryos at nc12 and nc14b stained by *in-situ* hybridization using a *sog* intronic riboprobe to examine effects on *sog* endogenous gene expression. Embryos are oriented with anterior to the left and dorsal upwards. In *hs-run* embryos, no *sog* expression is identified at nc12 as indicated by the red arrowhead (J); whereas at nc14b, *hs-run* embryos exhibit increases in *sog* levels and domain of expression relative to control (*hs-wt*), as indicated by the red arrowhead (K).
